# Book Reviews

**Published:** 1873-04-01

**Authors:** 


### Book Reviews.

The volume of Clinical Lectures by Prof. N. S. Davis, announcement of which will be found in our advertising columns, is now ready.

In order to give our readers an idea of the character and scope of the work, we copy the following table of contents:

Lecture I. — *Continued Fever.* — Cases of Typhus and Typhoid Fever.— Bad Effects of Cathartics. — Value of Strychnia in Certain Stages. — A Case of Typho-Malarial Fever.

Lecture II. — *Continued Fever.* — Cases Complicated with Emboli. — Bronchitis, Broncho-Pneumonia, Hypostatic Infiltration, Intestinal Ulceration, etc.

Lecture III. — *Cases of Periodical Fever.* — Chronic Ague and its Sequelæ.

Lecture IV. — *Rheumatic Fever.* — Acute Articular Rheumatism.—Muscular and Bronchial Rheumatism. — Rheumatic Inflammation of Spinal Nerves. — Intestinal Rheumatism. — Chronic Rheumatism, etc.

Lecture V. — *Scarlatina and Rubeola.* — Renal Dropsy.— Suppression of Urine.— Convulsions. — A Case of Rubeola.

Lecture VI. — *Respiratory Affections.* — Diphtheria.— Croup.—Chronic Bronchitis. — Rheumatic Bronchitis.— Asthma.— Pleurisy, etc.

Lecture VII.—*Pulmonary Tuberculosis.*— Cases Illustrative of the Incipient, Suppurative, and Excavated Stages. — Diagnosis. — Treatment.

Lecture VIII. — *Diseases of the Alimentary Tract.* — Gastritis. — Scirrhus at the Pyloric Orifice of the Stomach. — Chronic Inflammation of the Stomach. — Chronic Army Diarrhœa, Chronic Diarrhœa, Sequel of Typhoid Fever. — Acute Dysentery.

Lecture IX.—*Intestinal and Uterine Irritation.*— Bilious Colic.— Tobacco Enemas.— Hydrate of Chloral and Belladonna Enemas. — Inflammation and its Treatment.

Lecture X.—*Summer Complaints of Children.* — Diarrhœa. — Cholera Infantum, etc.

Lecture XI. — *Summer Complaints of Children.* — Their Pathology and Treatment.

Lecture XII. — *Dropsy.* — The Pathological Conditions that Give Rise to it. — Differences in Location and Progress.

Lecture XIII. — *Cases of Cardiac Disease.* — Diabetes Mellitus. — Anæmia and Anasarca. — Biliary Calculi.

Lecture XIV.—*Neuralgia.*—An Obscure Case. — Sciatica. — Neuralgia of the Rectum.

Lecturr XV.—*Nervous Affections.*— Spinal Irritation.—Hemiplegia.— Partial Hemiplegia—Paraplegia.—Progressive Locomotor Ataxia.

Lecture XVI.—*Affections of the Brain.*— Epilepsy. — Probable Softening of the Brain. —Chronic Hydrocephalus.

Lecture XVII. — *Cerebro - Spinal Disease.* — Symptoms, Progress, and Treatment.

Lecture XVIII. — *Cutaneous Diseases.* — Their Classification and Diagnosis. —Cases of Psoriasis.—Prurigo.—Porrigo.—Eczema, etc.

We have received the following books through Messrs. Jansen, McClung & Co., 117 State street, Chicago:

Diseases of the Ovaries: their Diagnosis and Treatment. By T. Spencer Wells. New York: D. Appleton & Co.

Wahler's Outlines of Organic Chemistry. By Rudolph Fittig, Ph.D., etc. Translated from the eighth German edition. With additions by Ira Rewson, M. D., Ph.D. Philadelphia: Henry C. Lea.

The Science and Art of Surgery, being a Treatise on Surgical Injuries, Diseases, and Operations. By John Eric Ericksen. A New edition, enlarged and carefully revised. 2 vols.

Will be reviewed in our next number.

